# A viral trigger for celiac disease

**DOI:** 10.1371/journal.ppat.1007181

**Published:** 2018-09-20

**Authors:** Judy J. Brown, Bana Jabri, Terence S. Dermody

**Affiliations:** 1 Department of Pathology, Microbiology, and Immunology, Vanderbilt University School of Medicine, Nashville, Tennessee, United States of America; 2 Department of Pediatrics, University of Pittsburgh School of Medicine, Pittsburgh, Pennsylvania, United States of America; 3 Elizabeth B. Lamb Center for Pediatric Research, Vanderbilt University School of Medicine, Nashville, Tennessee, United States of America; 4 Department of Medicine and Committee on Immunology, University of Chicago, Chicago, Illinois, United States of America; 5 Department of Microbiology and Molecular Genetics, University of Pittsburgh School of Medicine, Pittsburgh, Pennsylvania, United States of America; University of Florida, UNITED STATES

## Introduction

Celiac disease (CD) is an autoimmune enteropathy that occurs in genetically susceptible individuals exposed to dietary gluten. CD occurs in approximately 1 in 133 persons in the United States [1], although most are undiagnosed. Young children with CD present with diarrhea and malabsorption, but CD is also associated with extraintestinal autoimmune disorders, infertility, miscarriages, and cancer [2]. Ingestion of gluten is the most important environmental factor that correlates with CD [3]. Accordingly, current treatment strategies are centered on maintaining a gluten-free diet, which is challenging for many with CD [4]. Because of the increasing prevalence of CD [5] and the consequences of misdiagnosis, it is essential to better understand CD pathogenesis.

## Viruses and CD

Although 30–45% of the United States population has the CD risk alleles (HLA haplotypes DQ2 and DQ8), only 1% of the population develops the disease [1]. Therefore, unidentified triggers of CD must exist to cause the initial insult that breaks oral tolerance to gluten and establishes lasting pathogenic immune memory.

There are several clues that implicate infectious agents, particularly viruses, as triggers of CD. Viral infections often induce type 1 interferons (IFNs) [6], which break oral tolerance and precipitate development of CD in mice [7, 8]. In humans, treatment with IFNα can lead to CD [7]. Type 1 IFNs also form critical nodes in the network of CD susceptibility genes [3]. Finally, infections with adenovirus, enterovirus, hepatitis C virus, and rotavirus are associated with an increased incidence of CD [9, 10].

Epidemiological studies of children during the Swedish CD epidemic of 1987 to 1997 found that repeated neonatal infections were linked to CD onset (odds ratio [OD] = 1.52) [11]. A prospective study of at-risk children found that children infected with rotavirus had a higher prevalence of CD and that repeated infections intensified this effect (OD = 1.94 for one infection and OD = 3.76 for two or more infections) [10]. However, despite anecdotal and clinical implications that microbial pathogens act as triggers of CD, little is known about the mechanisms by which infectious agents evoke the disease.

## Reovirus breaks oral tolerance

During lymphocyte development, B and T-cell receptor diversity is required to mount successful responses against pathogenic microbes. Immune tolerance selects against B and T cells that express receptors that recognize self-antigen and thus could harm the host. In the intestine, a unique type of immune tolerance, known as oral tolerance, induces local and systemic unresponsiveness following oral feeding and prevents unnecessary immune responses to food proteins. Following antigen feeding, oral tolerance prevents delayed-type hypersensitivity (DTH) responses by inhibiting T-cell proliferation, cytokine production, and serum antibodies against the food protein [12, 13].

Normally, food proteins absorbed by the intestine are taken up by antigen-presenting cells in the lamina propria (LP) underlying the villus epithelium [14]. Oral tolerance is dependent on LP dendritic cells (DCs) that transport oral antigen to the draining mesenteric lymph nodes (MLNs) [15] and promote gut-homing T-cell responses [16]. T cells with suppressive functions, forkhead box P3 (Foxp3)^+^ regulatory T cells (T_regs_), inhibit inflammatory T-cell responses against food antigens during oral tolerance [17], a property that can be adoptively transferred to naïve animals and abrogated by the removal of these cells [18].

Oral tolerance to food antigens is dependent on intestinal DCs that express tolerogenic factors to promote antigen-specific T_reg_ responses [16]. However, intestinal DCs stimulate inflammatory CD4^+^ T cell responses against gluten in the intestinal mucosa of persons with CD [19]. Following ingestion of gluten, inflammatory, gluten-specific CD4^+^ T cells (T_H_1) license B cells to secrete anti-gluten and autoimmune antibodies and produce cytokines that mediate killing of intestinal epithelial cells (IECs) [3]. In turn, enterocyte destruction results in blunted intestinal villi and a failure to efficiently absorb food nutrients. The switch in DC state may result from changes in the intestinal environment. Such disruptions could be explained by high levels of inflammatory cytokines, including type 1 IFNs. Based on these observations, we hypothesized that viral infections of the intestine alter the immune response to oral antigens such as gluten and lead to development of CD.

Reoviridae family viruses are nonenveloped, double-stranded RNA viruses that infect humans frequently throughout their lifetime [20]. Mammalian orthoreovirus (reovirus) strains isolated from humans can infect mice via the oral route and activate innate immune pathways, like the related rotavirus [21]. Reovirus also stimulates type 1 IFNs [22]. Feeding mice ovalbumin (OVA) as a model antigen results in systemic tolerance to OVA, which is marked by induction of T_regs_ and absence of OVA-specific inflammatory T_H_1 cells [23, 24]. Peroral inoculation of reovirus strain T1L abrogates oral tolerance to OVA, as evidenced by a reduction in T_regs_ and promotion of OVA-specific T_H_1 cells [24]. Furthermore, HLA-DQ8–transgenic mice inoculated with T1L and fed gliadin, a proteolytic derivative of gluten, develop gluten-specific antibodies and a DTH response to gluten antigen, indicating that the mice do not establish tolerance to gluten [24]. Infection with T1L activates transglutaminase 2 [24], an enzyme that enhances CD immunopathogenesis [25]. Thus, reovirus can trigger inflammation to dietary gluten, which establishes a model of virus-induced CD.

In humans, reovirus infections are common during early childhood [20] when maternal immunity is waning and solid foods, including wheat cereals, are introduced into the diet. This also is the time at which children are most susceptible to developing CD [4]. Persons with CD express higher levels of reovirus antibodies compared with controls [24], raising the possibility that reovirus infection is linked to the development of CD in humans. Infection with rotavirus, another Reoviridae virus, correlates with the onset of CD in a longitudinal study [10]. However, no association was found in a subsequent cohort study [24]. Such discrepant results emphasize the importance of further clinical and mechanistic research to understand how viral infections trigger CD.

## Not all reovirus strains induce tolerance loss

**Fig 1:**
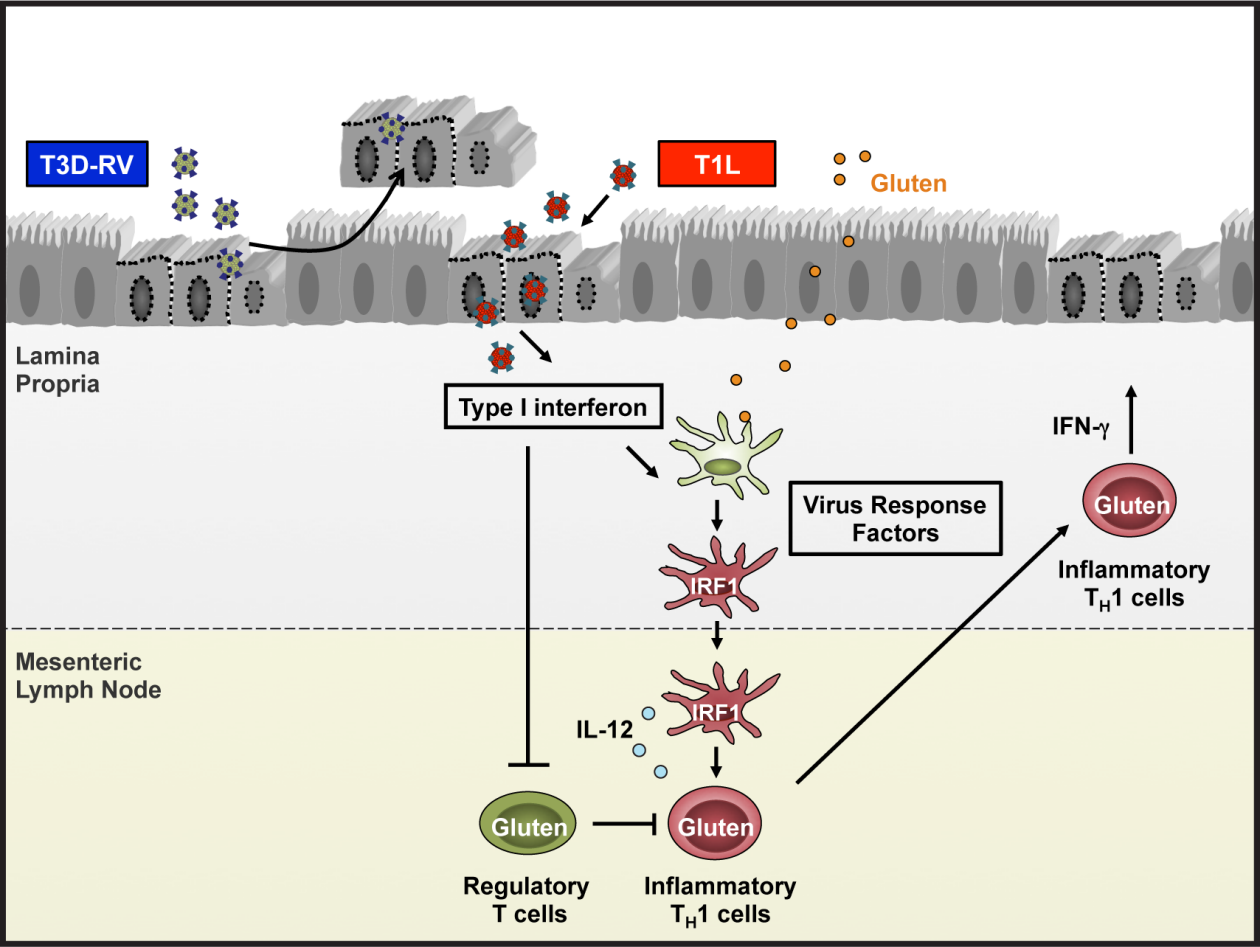
Model of reovirus strain-specific induction of celiac disease. Following peroral inoculation, reovirus T1L and T3D-RV infect the intestine. T3D-RV causes caspase-3 activation and intestinal epithelial cell sloughing, which subsequently leads to rapid viral clearance. T1L, however, subverts these antiviral responses to establish prolonged infection, triggering release of type 1 interferons and other virus response factors (yet to be defined) that induce IRF-1 expression in lamina propria dendritic cells. In the context of this inflammatory cytokine milieu, dendritic cells phagocytose new food antigen (such as gluten), traffic to the mesenteric lymph nodes, and secrete IRF-1–induced IL-12 to activate gluten-specific inflammatory T cells (T_H_1). Type 1 interferons up-regulated during T1L infection also inhibit regulatory T cells, leading to expansion of T_H_1 immunity to gluten in the development of celiac disease. IRF-1, interferon regulatory factor-1; T_H_1, gluten-specific inflammatory T cells.

The inhibition of oral tolerance by reovirus is strain specific [24]. Infection with reovirus T1L abrogates oral tolerance to fed antigen, as seen in CD, while infection with T3D-RV does not (Fig 1). Relative to infection with T3D-RV, T1L infection is associated with increased levels of inflammatory mediators, including type 1 IFNs and IFN regulatory factor-1 (IRF-1) [24], which are up-regulated in the intestinal mucosa of CD patients [26, 27]. Type 1 IFNs are not required for the differentiation of inflammatory food-specific T_H_1 cells. However, type 1 IFNs are required to inhibit conversion into Foxp3^+^ T_reg_ cells, suggesting that these cytokines, although dispensable for development of inflammatory food-specific T cells, inhibit tolerogenic processes [24]. IRF-1, a transcription factor implicated in multistage regulation of T_H_1 immune responses and antiviral immunity [28], is required to induce reovirus-mediated, OVA-specific inflammatory T_H_1 cells, likely via stimulation of IL-12 in LP DCs [24]. These results suggest that the switch from tolerogenic to inflammatory DCs results from viral stimulation of type 1 IFN and IRF-1 and that viruses producing higher levels of these factors are more likely to irreversibly disrupt immune homeostasis in the development of CD.

Differing levels of inflammatory cytokines induced by T1L and T3D-RV could be explained by a strain-specific capacity to infect the intestine. Although both viruses produce comparable titers in the intestine, Peyer’s patches, and MLNs 24 hours after peroral inoculation, T3D-RV is cleared more rapidly than T1L (with lower viral titers at 48 and 72 hours postinfection). Termination of T3D-RV infection is associated with activation of caspase-3, a marker of noninflammatory apoptotic cell death. Furthermore, T1L × T3D-RV reassortant viruses that induce limited apoptosis in the intestine display enhanced infection capacity, similar to T1L [29]. These results suggest that apoptotic death of IECs protects against enteric infection and that virus strains subverting this response have a replication advantage. We hypothesize that the prolonged infection capacity of T1L may stimulate greater levels and enhanced expression of inflammatory cytokines required to break oral tolerance to fed antigen (Fig 1).

## Future directions

Viral capacity to infect the intestine, avert host antiviral responses, and induce high levels of inflammatory cytokines may dictate whether a specific virus breaks oral tolerance. To test this hypothesis, T1L × T3D-RV reassortant viruses can be used to define the pathobiological properties associated with the abrogation of oral tolerance and identify specific viral gene products and functional domains that elicit such phenotypes. Although IRF-1 has been defined as a host factor required for the disruption of oral tolerance, other host factors have yet to be determined. Since apoptosis functions in accelerating clearance of reovirus in the intestine, studies using caspase inhibitors and mutant mice lacking apoptosis effectors should allow the role of apoptosis in prevention of reovirus-induced tolerance blockade to be clarified. It also will be important to understand the contribution of the intestinal microbiota in the differential host response displayed by T1L and T3D-RV. These studies will reveal underlying mechanisms by which viruses break oral tolerance to gluten in the development of CD.

To prevent virus-induced CD, we must first define the kinetics of infection associated with loss of oral tolerance. We hypothesize that viral infection must coincide with the introduction of a new food antigen such as gluten. To test this hypothesis, the timing of reovirus infection can be altered relative to the introduction of gluten to determine whether mice develop CD-like immunopathology. Additionally, mice can be sequentially infected with reovirus and other enteric viral pathogens to investigate the effect of multiple enteric infections on tolerance loss. Most relevant to the development of CD prophylaxis are experiments to determine whether vaccination can prevent loss of oral tolerance to gluten. Mice can be immunized with inactivated reovirus or viable reassortants lacking immunopathological properties prior to inoculation with T1L and introduction of dietary gluten. Taken together, these studies will provide rationale for prospective human trials to determine whether reovirus infection precedes the onset of CD and whether reovirus vaccination can prevent CD development.
